# A case report of melanotic neuroectodermal tumor of infancy complicated with congenital heart disease and hypothyroidism

**DOI:** 10.3389/fcvm.2022.924538

**Published:** 2022-11-10

**Authors:** Hua-Chu Zuo, Jin-Yue Huang, Xiao-Li Hu, Lin-Sheng Zhao

**Affiliations:** ^1^Department of Pathology, Tianjin Children’s Hospital (Tianjin University Children’s Hospital), Tianjin, China; ^2^Institute of Pediatrics, Tianjin Children’s Hospital (Tianjin University Children’s Hospital), Tianjin, China

**Keywords:** melanotic neuroectodermal tumor of infancy, congenital heart disease, hypothyroidism, case report, frequently encountered disease

## Abstract

To the best of our knowledge, thus far there are no reported cases of melanotic neuroectodermal tumor of infancy (MNTI) with multiple complications. In this case report, we describe the clinical phenotype of MNTI in a 9-month-old female infant associated with tetralogy of Fallot (TOF), a congenital heart defect, and congenital hypothyroidism (CH). Our study showed that the growth of MNTI was delayed by a lower dosage of levothyroxine (L-T4) that was prescribed to treat CH because of the presence of TOF, a severe congenital heart disease. However, the standardized dosage of L-T4 improved thyroid function but stimulated the rapid growth of MNTI. Our report demonstrated that treatment with L-T4 affects the progression of MNTI. Our findings demonstrated the role of thyroid hormone in MNTI growth and progression. Furthermore, our study suggested that the treatment of co-morbidities in children with MNTI requires careful consideration of their effects on the growth and progression of MNTI.

## Introduction

Melanotic neuroectodermal tumor of infancy (MNTI) is a rare, rapidly growing, benign, and pigmented tumor of neural crest origin that was first reported by Krombecher as congenital melanocarcinoma in 1918 (1). To date, nearly 500 MNTI cases have been reported worldwide. The mean age of onset for MNTI is 5 months and is mostly reported in male infants under 1 year of age (2). More than 90% of the MNTI lesions occur in the craniofacial region, especially in the maxillary (62.2%), cranial (15.6%), and mandibular (7.8%) regions; the other locations of MNTI lesions include the testes, epididymis, ovaries, uterus, and soft tissues of the extremities (3). The histopathological features of MNTI lesions indicate the presence of small rounded neuroblast-like cellular areas surrounded by large areas of melanin-containing cells that include a combination of epithelial, neural, and melanocytic cells. The MNTI lesions show rapid, infiltrative growth. Surgical resection is the conventional treatment for MNTI. However, the recurrence rate is 10–60% because of anatomical limitations and incomplete excision to prevent damage to the adjacent tissues (4). The differential diagnosis of MNTI includes other small blue round cell tumors in children, such as neuroblastoma, Ewing’s sarcoma, adenoid rhabdomyosarcoma, melanoma, and clear cell sarcoma (CCS) of soft tissue. Previously published studies mainly focused on the histopathological and immunohistochemical differences between the lesions in different anatomical sites, the scope of surgical resection treatment, and age-related characteristics (5, 6). However, so far, none of the reports have reported a variety of complications in the same case. Therefore, in this case report, we describe a 9-month-old female infant with a single, painless, non-ulcerated, pigmented, fast-growing, cranial (top of frontal) neoplasm with features that are as described previously for MNTI lesions. The patient was also diagnosed with TOF, a severe congenital heart disease, and CH after birth. Low-dose L-T4 treatment suppressed MNTI growth, but the tumor size increased significantly after cardiac surgery and standardized L-T4 treatment. This suggested that precautions and careful follow-up are necessary for treating MNTI patients with co-morbidities involving thyroid dysfunction and cardiac malformations. Moreover, genetic testing is recommended for cases with multiple organ diseases to determine the potential underlying genetic abnormalities. In cases without evidence of mechanisms promoting MNTI growth and development, our study suggested that medications reducing thyroid function may control tumor growth.

## Case description

This case described the clinical characteristics of a 9-month-old girl with a 6-month history of growing mass in the left frontal cortex with faster growth in the recent 3 months. She was diagnosed with TOF and CH after birth and underwent radical surgery at the age of 6 months. The patient was given a reduced L-T4 dose for CH treatment because of the underlying TOF. After surgery, the dosage was gradually increased.

### Physical examination of the melanotic neuroectodermal tumor of infancy

The tumor was present on the left forehead and showed basal fixation. It was hard, about 3 cm × 4 cm in size, and did not show any signs of neurologic involvement. The skull did not show asymmetry, cutaneous lesions, or inflammation. Neck mass was not observed. There were no symptoms of chronic hypoxia, such as paroxysmal cyanosis and clubbing fingers. The physical growth and development of the infant were normal and in range for her age group.

### Brain computed tomography scan details

**FIGURE 1:**
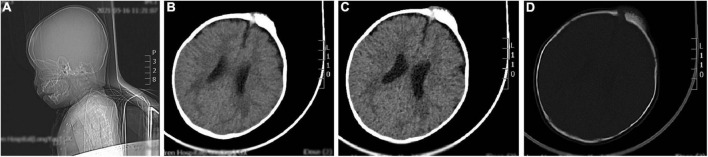
A representative image of the head CT scan shows the irregular bone structure with mixed density mass on the left side of the cranial plate in the median frontal area, slightly reduced density in the bilateral parietal lobes, widening of the ventricles and extracerebral space, and thickening of the septal sinus mucosa. **(A)** Sagittal view shows a mass in the frontal area. **(B,C)** Soft tissue window at the central level of the lateral ventricle show a high-density mass in the left frontal region. **(D)** Bone window at the central level of the lateral ventricle: the bony structure of the left cranial plate in the middle frontal region is irregular, and there is a mixed density mass shadow.

The brain CT scan showed irregular bone structure with mixed density mass on the left side of the cranial plate in the forehead, slightly reduced brain mass density in the bilateral parietal lobes, and wider ventricles and extracerebral spaces as shown in Figure 1.

### Cardiac ultrasound characteristics

**FIGURE 2:**
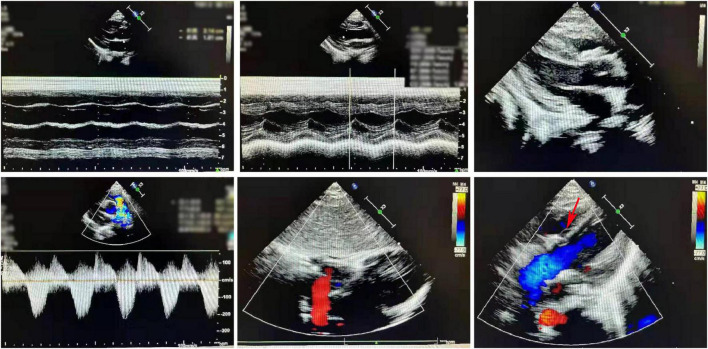
A representative image of the cardiac ultrasound (postoperative) shows the normal internal diameter of each chamber, low septal motion amplitude, and strong plaque echoes around the septum. The right ventricular outflow tract is not obstructed. The muscle bundles are not hypertrophic. The internal diameter of the pulmonary artery was normal.

The postoperative cardiac ultrasound (Figure 2) showed that the inner diameter of the cardiac cavities was within the normal range. However, the motion amplitude of the ventricular septum was reduced. The ventricular septum and the left ventricular posterior wall were not thick. The left ventricular posterior wall showed normal motion. The atrial septum was continuous and complete. A strong patch echo was observed around the ventricular septum. The surrounding tissues did not show any obvious cracks. The morphological structure and the opening and closing movements of the valves were normal. The right ventricular outflow tract was not obstructed. A hypertrophic muscle bundle was not detected. The inner diameter of the pulmonary artery was normal (Figure 2). Doppler examination did not detect any shunt signal at the systolic ventricular level. The peak forward flow velocity of the pulmonary artery flow was 2.2 m/s (Figure 2).

### Thyroid function tests

The thyroid function was normal at admission.

### Surgical removal, histopathology, and immunohistochemical details of the melanotic neuroectodermal tumor of infancy

After admission, the left frontal tumor was resected under general anesthesia. After satisfactory anesthesia, the skin in the surgical area was routinely disinfected. An incision was made through the skin tissue, capillary tendon membrane, and the periosteum at the top of the frontal curvature to expose the skull. The skull in the surgical area was abnormally elevated, dark red in color, and poorly defined in comparison with the surrounding normal skull. Second, the abnormal skull was bitten, with a loose texture and poor blood supply. The abnormal skull was removed by grinding and drilling, and the cap-like aponeurosis, subcutaneous tissue, and skin were sutured. The wound was pressure dressed. The operation went smoothly without much bleeding. The patient was discharged after suture removal.

**FIGURE 3:**
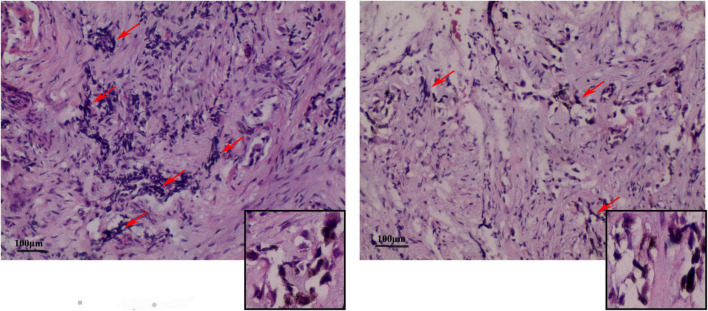
Representative image (× 100) shows the histopathological analysis of the tumor tissue. The tumor tissue consists mainly of neuroblastoma-like cells and melanin-containing epithelial cells. **(Left)** The small round or square and primitive neuroblastoma-like cells are large and contain melanin granules in the cytoplasm of the epithelial cells. The deep-stained and shrunken neuroblastoma-like cells are mostly arranged in nests with extruded nuclei. **(Right)** Pigmented epithelioid cells with vacuolated nuclei are observed in the large area surrounding the neuroblastoma-like cells. Some areas are mainly composed of pigmented epithelioid cells arranged in sheets, nests, or beam bundles, with dense fibrous connective tissue between the nests of cells, and some areas can be seen as bone trabeculae with visible nuclear schizograms (0–2 per 10 high magnification fields).

Under the postoperative pathological microscope (Figure 3), most of the lesion tissue was hyperplastic and fibrous with abundant new bone trabeculae. Few bone trabeculae showed osteoblasts. Irregular epithelioid cell clusters were scattered in the hyperplastic fibrous tissue. Melanin particles were observed in some cells. In between fibrous tissues, small blue cells with obvious multi-focal compression were observed.

**FIGURE 4:**
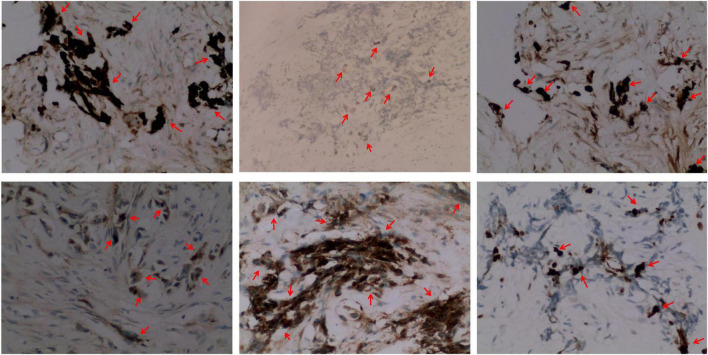
Representative immunohistochemical staining images (× 200) (from **left** to **right)** of the MNT1 tumor sections for cytokeratin (CK, epithelioid +), S-100 protein (epithelioid +), melanoma antibody (HMB45, epithelioid +), neuron-specific dilute alcoholase (NSE, small round cell +), synaptophysin (Syn, small round cell +), and Ki67. Nearly, 15% of the nuclei were Ki-67 positive.

Immunohistochemistry of the lesion showed cytokeratin (CK)-positive epithelial cells, synaptophysin (syn)-positive small blue cells, S-100-positive, HMB45-positive, CD99-negative, CD3-negative, NSE-positive, ERG-positive vascular endothelium, and 15% Ki67-positivity (Figure 4). Based on these findings, the lesion was diagnosed as MNTI.

**FIGURE 5:**
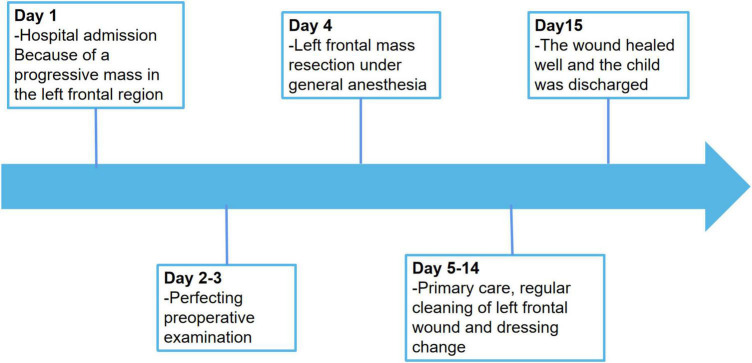
Timeline of the patient’s clinical journey.

The postoperative outcome of the patient was good and did not show any additional neurologic impairment. Chemotherapy and/or radiotherapy were not required. The timeline of the patient’s clinical journey is shown in Figure 5. Brain MRI at the 12-month follow-up did not show any tumor recurrence.

## Discussion

MNTI is a rare tumor of neural crest origin that is associated with increased urinary excretion of vanilloid acid (7). The biphasic population of melanocytes and primitive neuroectodermal cells in MNTI are both linked to the neural crest cells (NCCs), which are unique migratory pluripotent stem cells in the vertebrates that are required for the generation of critical components of the craniofacial skeleton, melanocytes, and ganglia of the peripheral nervous system. The generation, migration, and differentiation of NCCs are tightly regulated. Therefore, aberrant development of the NCCs causes embryonic developmental abnormalities and is associated with a spectrum of disorders called neurocristopathies (NCPs). The majority of the known craniofacial developmental defects are associated with alterations in NCC development. Moreover, craniofacial birth defects account for almost one-third of all congenital birth defects reported in humans (8). Neuroblastoma is the most common cancer in infants who are younger than 1 year old and accounts for 1 in every 100,000 births (9). Reed (10) classified NCPs based on the presence or absence of accompanying tumors into developmental abnormalities (dysplasias), neoplasms, and combination of malformations and tumors. This classification was too simple and underestimated the incidence of neural crest disease because of the high variability of the NCPs and inaccurate diagnosis of the minor phenotypic malformations.

Clinically, MNTI is described as single, painless, non-ulcerated, pigmented, and fast-growing neoplasms. In this case-report, a fast-growing mass appeared in the left frontal cranial plate, after the child underwent radical surgery for TOF, a congenital heart disease, at the age of 6 months. This was consistent with the morphological characteristics of MNTI. The pre-operative CT image showed irregular bone structure and irregular soft tissue mass on the left side of the cranial plate in the middle of the forehead, and non-uniform density and “needle-like” periosteal reaction in the parietal bone (11). MNTI neoplasms are mainly composed of melanin-containing epithelial cells and small round neuroblastoma-like cells. MNTI is highly heterogeneous. Therefore, immunohistochemistry is an important tool for the diagnosis of MNTI. The epithelial-like cells in MNTI show higher levels of CK and HMB-45, and reduced S-100 protein levels. The small round neuroblastoma-like cells in MNTI lesions show high expression levels of CD56 and synaptophysin. The neuron-specific enolase is observed in both types of cells. In this study, the immunohistochemical staining data for the patient was consistent with previous reports. However, immunohistochemistry does not reflect the biological behavior of the tumor.

MNTI biopsies is susceptible to misdiagnosis as neuroblastoma. However, neuroblastoma lacks epithelial cells with melanin and is negative for CK and HMB45. The differential diagnosis of MNTI includes other small round blue cell tumors in children such as Ewing sarcomas, adenoid rhabdomyosarcomas, melanomas, and soft tissue CCSs. Ewing sarcoma shows varying degrees of neuroectodermal differentiation and includes morphologically similar small round blue cell tumors and common chromosomal translocations. The Ewing sarcoma family of tumors (ESFTs) are characterized by chromosomal translocations involving one of the several genes of the ETS family (*FLI1, ERG, ETV1, ETV4*, and *FEV*) and the *EWS* gene called *Ewing sarcoma breakpoint region 1* (*EWSR1*) on chromosome 22q12. The most commonly reported chromosomal translocation is t(11;22) (q24;q12), which involves the *EWS* and *FLI1* genes and is observed in 83% of cases with ESFTs. This occurs through an in-frame fusion of the *EWS* gene at 22q12 and the *FLI1* gene at 11q24 resulting in the formation of the *EWS-FLI1* fusion gene product (12). The tumors with the *EWS/FLI-1* fusion gene are characterized by small round cells that are arranged closely to form a Homer-Wright daisy-chain structure and the membrane-positive staining for the MIC gene product, CD99, in 90% of the cases. CD99 staining plays an important role in the diagnosis of Ewing sarcoma. However, Ewing sarcomas lack melanin-containing epithelial cells.

Adenoid rhabdomyosarcomas show morphological similarities with MNTI including adenoid or solid nested structures formed by naive small round cells with specific chromosomal abnormalities. The most common chromosomal translocation in adenoid rhabdomyosarcomas is the t(2;13) (q35; q14) translocation that generates the *PAX3/FOXO1* fusion gene. The fusion proteins in adenoid rhabdomyosarcomas act as transcriptional activators and regulate myogenic differentiation by altering cellular growth, motility, differentiation, and apoptotic pathways that promote tumor development and metastasis. The nuclear schwannomas show diffused positivity for MyoD1 and myogenin and mostly negative staining for synaptophysin and NSE. In MNTI, transverse myoblast differentiation is occasionally seen, but not in small biopsies.

Since epithelial cells in MNTI neoplasms produce melanin, they need to be differentiated from melanomas. Melanomas are generally heterotypic and can be screened using the S-100 protein. However, the S-100 protein cannot be used as a confirmatory indicator because of its low specificity. Melan A, HMB45, and tyrosinase show higher specificity and varying sensitivity in melanomas, whereas CK and neuroendocrine markers are negative. Furthermore, in the melanomas, the Ki-67 positivity index and cyclin D1 expression levels were high and did not decrease with lesion depth. Therefore, for comprehensive diagnosis of MNTI, two or three of the above-mentioned markers are combined with the S-100 protein index (13).

A slow-growing painless tumor mass is characteristically observed in more than half of the patients with primary soft tissue CCS. In patients with CCS, immunohistochemistry analysis shows strong positivity for S-100, HMB45, melanA, and Vimentin. CCS is also associated with the t(12;22) (q13;q12) translocation and lack of neuroblastoma-like cells (14).

In this case, intraoperatively, the endplate of the milled bone flap showed a “pinprick-like” protrusion that was infiltrative and closely associated with the deep dura mater. This suggested that preoperative imaging was important for the diagnosis of MNTI to provide a reference for guiding the extent of surgical resection of the lesion and the surrounding tissues.

Surgical excision of the lesion is the treatment of choice for patients with MNTI. The prognosis for MNTI is generally good with a postoperative recurrence rate of approximately 15–45% mostly due to incomplete resection of the primary lesion (2). In 3% of MNTI cases, metastasis to the lymph nodes or the central nervous system has been documented (15, 16). Some studies suggest that the resection of the primary lesion should be expanded to remove the majority of the tumor that invades the surrounding tissues to decrease the rates of recurrence. However, it is difficult to determine the scope of expanding the resection site because the tumor originates mostly in the head and face and represents obstacles from anatomical structures (17). Adjuvant chemotherapy or combined radiotherapy is recommended as an alternative to surgery for patients with MNTI when total resection of the tumor is not possible or in cases with contraindications to surgery (18). Furthermore, age at diagnosis is a risk factor for recurrence. Infants diagnosed within the first 2 months of life have the highest likelihood of recurrence within 6 months and the shortest disease-free survival; infants diagnosed at 4.5 months or older have the lowest risk of recurrence; and those diagnosed between 2 and 4.5 months have an intermediate probability of recurrence (5). In this case, although the child was diagnosed at 6 months of age, the previous history of CH and congenital heart disease raised concerns regarding other co-morbidities and the risk of recurrence within 6 months. During the resection of the left frontal mass, we observed characteristic pigmentation of MNTI and localized “pinprick-like” reaction of the skull and adhesion of the tumor tissue on the dura mater. Although the resected tumor could have been frozen rapidly and sent for pathological examination, the extent and severity of surgical trauma would have affected the pathological findings. The tumor tissue was too close to the bone margin. Although the bone margin in the bone window did not show any obvious abnormality by visual observation, the skull was enlarged and 1 cm of tissue around the bone window was removed. The affected dura was also removed, and a dural patch was applied to repair the dural defect. The results of the 12-month postoperative follow-up showed that the child was in a good condition without any recurrence of the tumor mass *in situ* despite the choice to expand the surgical resection area, which increased the tissue trauma to the child. Therefore, although MNTI is a benign tumor, it is characterized by rapid growth and confers destructive effects on the adjacent tissues. The incomplete excision of the primary lesion because of anatomical constraints may lead to local recurrence. Therefore, early diagnosis and treatment are necessary. Moreover, the impact of other comorbidities or congenital developmental malformations needs to be observed during treatment and follow-up. The most effective treatment is complete resection of the lesion and the surrounding tissues that may be invaded by the tumor.

The aggressive course and poor prognosis of MNTI are based on the following prognostic indicators: nuclear schistosomes seen in more than 2 out of 10 high power fields (HPFs); greater than 25% Ki67 positive index; CD99 expression status; higher proportion of neuroblastoma-like cells compared to the epithelioid cells (6). In the present case, Ki67 positivity suggested rapid growth of the tumor and the risk of invasion. However, nuclear schistosomes in 0–2 out of 10 HPFs, negative CD99 staining, Ki-67 positive index of 15%, and appropriate ratio of the neuroblastoma-like cells to the epithelioid cells suggested a good prognosis for this child. The results of the 12-month postoperative follow-up showed the absence of *in situ* recurrences and confirmed the prognosis of the immunohistochemical index.

Characteristic cytogenetic or molecular abnormalities have not yet been identified in MNTI. The inhibition of Wnt and Shh signaling in the chicken embryos triggers craniofacial malformations and congenital heart disease by altering their migration and survival of NCCs and reducing the number of cranial and cardiac NCCs (19). Furthermore, mutations in the mouse and human *Pax3* genes lead to a spotted (Splotch) mutant phenotype and Waardenburg type I syndrome, respectively; the pure Splotch embryos show severe neural crest cell (NCC) defects with complex phenotypes such as spina bifida, extracerebral malformations, and cardiac outflow tract abnormalities (20). Pax3 expression is upregulated in glioblastomas, neuroblastomas, melanomas, rhabdomyosarcomas, and gastric cancer (21). Liang et al. (22) reported that Pax3 overexpression promoted human glioma cell proliferation, survival, and cell cycle progression by altering the expression levels of Wnt signaling proteins, such as β-catenin, Myc, VEGF, cyclin D1, MMP7, and Wnt1. Several transcription factors and signaling pathways synergistically regulate the complex developmental process of the NCCs. Therefore, aberrations in any of these regulatory proteins can affect the formation, migration, and differentiation of the NCCs, and promote the generation of neural crest-like progenitors.

Tetralogy of Fallot is the most common form of cyanotic congenital heart disease that accounts for 3–5% of all congenital heart diseases; it has a worldwide prevalence of about 0.05% and is most common in Asia (23). The embryologic pathogenesis of the tetralogy of Fallot is complex and involves aberrant neural crest cell migration, mesenchymalization of endothelial cells, and cardiomyocyte differentiation. Notch l and Notch 2 are expressed in specific cell populations of the neural crest-derived outflow tract and the epicardium, and play a key role in the cell-autonomous growth and differentiation of the cardiac neural crest precursors into the SMCs; defects or differential changes in some of these loci can cause congenital heart defects (24). In patients with tetralogy of Fallot, *NOTCH1* gene mutations are detected in nearly 4.5% of cases and are considered the predominant causative gene; mutations in the *NOTCH1* and *JAG1* genes in the Notch signaling pathway are associated with congenital heart disease (25). In this case, MNTI was associated with the manifestation of congenital heart disease and CH. Therefore, mutations and abnormalities in the genetic regulation network (GRN) of the NCCs should be investigated in such cases. Genetic testing of the co-morbidities can add more clinical phenotypes to the spectrum of NCPs and related diseases.

CH is a common disorder of the endocrine system in newborns and infants that can severely impair mental and physical development in children. The worldwide prevalence of CH is 20–200 per 100,000 and 85% of these cases are caused by abnormal thyroid differentiation, migration, or growth (hypothyroidism), and resistance against the thyroid stimulating hormone (26). Thyroid development, differentiation, and migration to their final location in the fetal tissues are regulated by transcription factors such as the paired box gene 8 (PAX8), thyroid transcription factor 2 (TTF2, also known as FOXE1), and the NK2 homeobox 1 (NKX2.1) (27). Several genes implicated in thyroid dysplasia are also associated with abnormalities in other tissues and organ development. It has also been shown that the migration and differentiation of the NCCs are involved in the formation of endocrine glands such as the thyroid. The migration and differentiation of the NCCs are regulated by a variety of signaling proteins such as the retinoids, fibroblast growth factor (FGF), endothelins, and the members of the Wnt signaling family (28). During fetal and neonatal development, deficiency of thyroid hormones impaired proliferation, migration, and differentiation of the thyroid-sensitive neurons resulting in the development of CH that caused neurological impairment, growth retardation, and mental retardation. Children with CH exhibit significant lag in visual, language, and fine motor development, and the symptoms are irreversible. *DUOX2* gene mutations are the most common cause of thyroid hormone synthesis disorder in the Chinese population (27). In this case, a previous ultrasound examination showed a normal location of the thyroid gland and excluded ectopic factors. However, genetic testing was not performed in this case. Therefore, the clinical features and etiology of the congenital thyroid defect could not be clarified.

Once CH is diagnosed, treatment should be administered immediately. L-T4 is the first choice of treatment for CH. However, in patients with severe congenital heart disease, the initial dose of L-T4 should be reduced. In this case, TOF, severe congenital heart disease was diagnosed soon after birth in combination with CH. The MNTI tumor did not show any significant increase in size during reduced L-T4 therapy. However, the tumor size was significantly increased after cardiac surgery and standardized L-T4 treatment. This suggested that the reduction of L-T4 because of TOF affected the complete recovery from hypothyroidism but retarded the growth of the tumor. After congenital heart surgery, the standardized use of L-T4 improved the recovery of thyroid function but accelerated tumor growth. Age-related growth of the MNTI tumor cannot be ruled out in this case. However, this case also suggests that MNTI tumor growth may be associated with adequate treatment of CH. Future studies of MNTI cases at a later stage would shed more light on the potential dysmorphic syndromes or neurodevelopmental disorders associated with MNTI. Furthermore, advances in genetic and other biochemical tests would aid in early prenatal diagnosis and therapeutic management.

## Conclusion

Congenital heart disease and/or congenital hypothyroidism are common complaints in children. However, their association with congenital neoplastic disorders is not clear. Our study shows that the growth and development of MNTI neoplasms require comprehensive and constant monitoring. Moreover, therapeutic interventions to co-morbidities may result in the progression of MNTI tumor growth. Genetic factors are the major risk factors for NCPs. Furthermore, large phenotypic heterogeneity and etiological homogeneity of the NCPs can significantly challenge unraveling the underlying pathogenic mechanisms of NCC development. Therefore, precise elucidation of the pathogenic mutations in the NCPs will help the development of targeted therapeutic and preventive tools.

## Data availability statement

The original contributions presented in this study are included in the article/supplementary material, further inquiries can be directed to the corresponding authors.

## Ethics statement

The studies involving human participants were reviewed and approved by the Medical Ethics Committee of Tianjin Children’s Hospital. It belongs to Tianjin Children’s Hospital. Written informed consent to participate in this study was provided by the participants’ legal guardian/next of kin. Written informed consent was obtained from the individual(s), and minor(s)’ legal guardian/next of kin, for the publication of any potentially identifiable images or data included in this article.

## Author contributions

H-CZ: methodology, formal analysis, investigation, and writing—original draft. J-YH: writing—original draft, resources, and visualization. L-SZ: resources, writing—review and editing, project administration, and funding acquisition. X-LH: conceptualization, data curation, writing—review and editing, and supervision. L-SZ and X-LH: responsible for the study described in this manuscript. All authors contributed to the article and approved the submitted version.
